# Transformation of Tertiary Benzyl Alcohols into the Vicinal Halo-Substituted Derivatives Using *N*-Halosuccinimides

**DOI:** 10.3390/molecules21101325

**Published:** 2016-10-02

**Authors:** Njomza Ajvazi, Stojan Stavber

**Affiliations:** 1 Department of Physical and Organic Chemistry, Jožef Stefan Institute, Jamova 39, 1000 Ljubljana, Slovenia; njomzaajvazi@hotmail.com; 2 Jožef Stefan International Postgraduate School, Jamova 39, 1000 Ljubljana, Slovenia; 3 Centre of Excellence for Integrated Approaches in Chemistry and Biology of Proteins CIPKeBiP (Seat at Jožef Stefan Institute), Jamova 39, 1000 Ljubljana, Slovenia

**Keywords:** halohydrins, halogenation, water, tertiary alcohols, *N*-halosuccinimides

## Abstract

The efficiency of direct conversion of tertiary alcohols bearing a β-hydrogen atom to vicinal halohydrins—chlorohydrins and bromohydrins—under green reaction conditions was tested preliminarily on model tertiary benzyl alcohols. Tertiary alcohols were successfully directly halogenated to vicinal halohydrins with *N*-halosuccinimide in aqueous media. The efficiency of the reaction in water was significantly improved in the presence of sodium dodecyl sulphate as the surfactant.

## 1. Introduction

Green chemistry, defined as “the invention, design, and application of chemical products and processes to reduce or to eliminate the use and generation of hazardous substances”, was founded by 12 basic principles nearly 20 years ago [1]. It became the general trend in chemistry, organic chemistry in particular, and has also had significant implementation in the pharmaceutical industry [2]. As organic solvents used in synthesis, isolation or purification protocols have had considerable impact on the green profile of particular processes, looking for alternatives for most applied solvents and evaluating their green characteristics is of great interest to academics as well as the industrial chemistry community [3,4,5,6,7]. Water is one of the most attractive alternatives for these tasks and the use of water as the reaction medium is no longer an unknown approach. The incompatibility of organic substrates with water as a result of hydrophobic interactions could represent a potential obstacle to performing organic reactions in water. Reactions in micellar systems have considerably extended organic chemistry in water and extraordinary selectivity and efficiency can be achieved through the addition of inexpensive amphiphiles that self-associate into micelles, thus accommodating insoluble organic compounds within hydrophobic cores and very often accelerating the rate of reaction. Investigation of organic reactions in aqueous reaction media has thus been very intense in last two decades [8,9,10,11,12].

Halohydrins are versatile building blocks and target intermediates for the synthesis of many bioactive molecules [13,14,15]. Methods for their synthesis have been comprehensively reviewed [16,17,18]. Behind ring opening of epoxides by hydrogen halide (HX), aldol type condensations, and reduction of α-haloketones, electrophilic halo-addition to alkenes in the presence of water is the most common approach to the preparation of these valuable compounds. In the last decade some attractive approaches to this kind of transformation of alkenes have been reported, such as *N*-halosuccinimide mediated reactions in water in the presence of β-cyclodextrin [19], ionic liquids [20], thiourea [21] or ammonium acetate [22] and use of H_2_O_2_/HBr system in water [23]. In our continuing interest in the green chemical approach to halotransformation of organic compounds, particularly those “in” or “on” water [12,24,25,26,27,28,29,30,31,32] we now report a new method for the preparation of vicinal chloro- or bromohydrins by *N*-halosuccinimide mediated halogenation of tertiary alcohols.

## 2. Results and Discussion

**Scheme 1:**
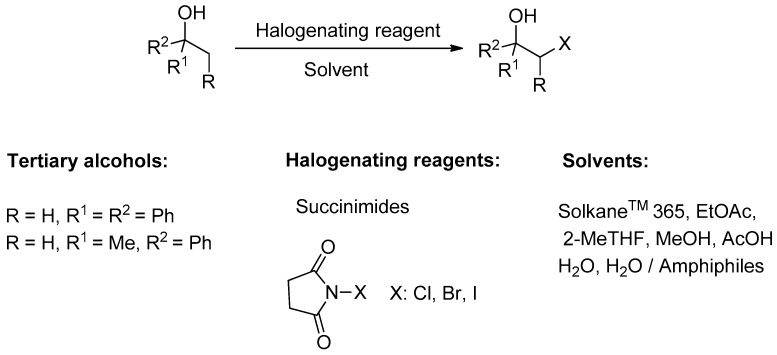
Direct conversion of tertiary benzyl alcohols into chloro-, bromo- and iodohydrins. Thesis and concept.

On the basis of our original discovery that fluorohydrins could be directly obtained by fluorination of tertiary alcohols by Selectfluor^TM^ F-TEDA-BF_4_ in MeCN solution [33], as well as by micellar system-mediated conversion in water [34], we wondered if the related transformation could also be realized for the synthesis of chloro- or bromohydrins. The basic thesis and the concept of its evaluation are given in Scheme 1.

According to our experiences documented in reference [33], benzyl alcohols bearing at least one vicinal hydrogen atom are the best choice for this transformation, we chose 1,1-diphenylethanol and 2-phenyl-2-propanol as the basic model compounds. *N*-chloro- (NCS), *N*-bromo- (NBS) and *N*-iodosuccinimide (NIS) were selected as halogen atom transfer reagents, while the list of environmentally accepted solvents [3,4,5,6,7] from aprotic Solkane^TM^ 365 to Me-THF, protic MeOH and AcOH, and finally water was chosen as reaction medium candidates. According to our experiences described in references [33,34] we assumed that secondary benzyl alcohols are not convenient candidates for this type of transformation. This assumption was confirmed also in the present case since reactions of several secondary benzyl alcohols tested gave very complex reaction mixtures.

From the data collected in Table 1, it can be seen that aprotic reaction media seems to be inconvenient for the halo-transformation of target tertiary alcohol **1**, since in 1,1,1,3,3-pentafluorobutane (Solkane^TM^ 365) or ethyl acetate none or low transformation of starting material **1** was observed (entries 1–4). On the onther hand, when the reactions were performed in a mixture of EtOAc/H_2_O we observed quantitative conversion of starting material **6** into the eliminated product **4** as the sole product (entries 5 and 6). In 2-methyltetrahydrofuran only dehydration to 1,1-diphenylethene (**4**) took place (entries 7 and 8).

In the case of performing the reactions in acetic acid, the quantitative conversion of **1** was observed using NCS (entries 9 and 11), and the formation of 2-chloro-1,1-diphenylethene (**3a**) was established as the main product, accompanied with a small amount of the eliminated product **4**, whilst in the case of reaction using NBS, the formation of the addition-elimination product **3b** was observed as the sole product, also after the shorter reaction time (entries 10 and 12). The decrease of the reaction temperature to 70–75 °C, in the case of using NCS, provided efficiently and selectively 2-chloro-1,1-diphenylethene (**3a**) in quantitative yield (entry 13), while in the case of using NBS, it afforded 2-bromo-1,1-diphenylethene (**3b**) in lower yield accompanied with the formation of 1,1-diphenylethene (**4**) (entry 14). When the reaction was carried out at 40 °C, using NCS or NBS, no conversion of starting material (**6**) occurred (entries 15 and 16).

On the contrary, in the case of reactions using NIS, the formation of complex reaction mixtures was observed.

In the case of reactions in pure methanol (Table 2) we found quantitative conversion of starting material **1** and the formation of the vicinal halomethoxy derivative **5a** (entry 1) as the main product using NCS, while in the case of the reaction using NBS, the related product **5b** was found as the minor product and alkene **4** as the main one (entry 2). The addition of water to the reaction media considerably decreased the selectivity of the transformation (entries 3–6).

When we treated 2-phenyl-2-propanol (**6**) with NCS or NBS in the above-mentioned solvents, the formation of very complex reaction mixtures took place in all cases, whilst using an AcOH/H_2_O mixture as solvent, the efficient and selective conversion to 2-halo-2-phenylpropan-2-oles could be achieved (entries 7 and 8, Table 2).

Water is one of the best alternatives to organic solvents [35]. It is naturally occurring, abundantly available, nontoxic, nonflammable and inexpensive. Although all natural processes occur in aqueous media, using water in the synthetic organic chemistry has never been popular. The reasons for this are the limited solubility of organic molecules in water which leads to lower rates or/and yields, and the hydrolytic instability of some reagents, products and catalysts. The major breakthrough in using water as a medium for organic reactions occurred with the studies on the Diels-Alder reaction done by Breslow in 1980 [36]. These studies concluded that organic reactions proceed well, or even better, in aqueous media, in comparison with organic solvents.

Initially, we checked the course of the reaction of 1,1-diphenylethanol (**1**) with *N*-halosuccinimides (NXS) in pure water and the results are given in Table 2. Since 1,1-diphenylethanol (**1**) and NXS are insoluble in water, the homogeneity of the reaction mixture was observed to be very low. The conversion of starting material **1** in the case of the reaction using NCS was thus found to be low (entry 9), while the NBS-mediated transformation gave considerably higher efficiency (entry 10) accompanied by 2,2-dichloro-1,1-diphenylethanol [37] or 2,2-dibromo-1,1-diphenylethanol [38] which are known compounds based on ^1^H-NMR spectra of isolated crude reaction mixture, but we believe that in both cases better results could be obtained. We further preliminarily investigated the reactions of 2-phenyl-2-propanol (**6**) with *N*-halosucinimides in pure water and the results are collected in Table 2. The conversion of starting material **6** in the case of the reaction with NCS was found to be quantitative, resulting in the formation of the vicinal halohydrin **7a** as the sole product (entry 11), while in the reaction with NBS, quantitative conversion of starting material **6** was observed, providing the vicinal halohydrin **7b** as the main product (due to the instability of the compound under chromatographic conditions, purification was unsuccessful), accompanied by the formation of eliminated product **8** (entry 12).

**Figure 1:**
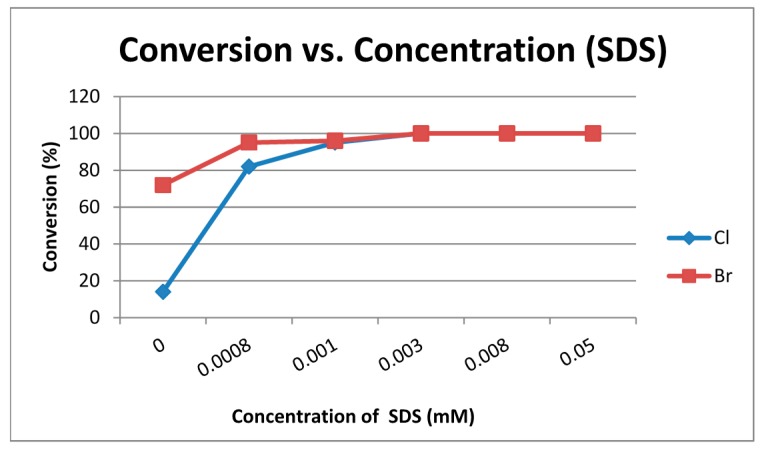
Effect of the added amount of sodium dodecyl sulphate (SDS) amphiphile on the efficiency of the reaction of 1,1-diphenylethanol (**1**) with *N*-halosuccinimide (NXS).

In order to improve the efficiency of this transformation, in the continuation of our work we tested if the addition of a surfactant had an effect on the course of the reaction. We chose sodium dodecyl sulfate (SDS) as the amphiphilic surfactant and reactions were performed with different surfactant concentrations in the aqueous media. Results are given in Table 3. The influence of the concentration of amphiphile on conversion of 1,1-diphenylethanol (**1**) in water with NCS and NBS is graphically presented in Figure 1 and it could be established that the catalytic effect of SDS amphiphile in the case of NCS and NBS-mediated reaction, reached its maximum efficiency at a concentration of 3 mM or higher. It was found that the choice of surfactant had a significant influence on the yield and the reaction time; namely, the addition of SDS increases the yields and reduces the reaction times.

Moreover, we checked the impact of SDS as the amphiphilic surfactant on the course of the reaction of 2-phenyl-2-propanol (**6**) with NCS and NBS and no effect was observed in either case. We further checked the reactions of both tested alcohols with NXS under solvent-free conditions but only starting material was recovered after 24 h at 70–75 °C.

**Scheme 2:**
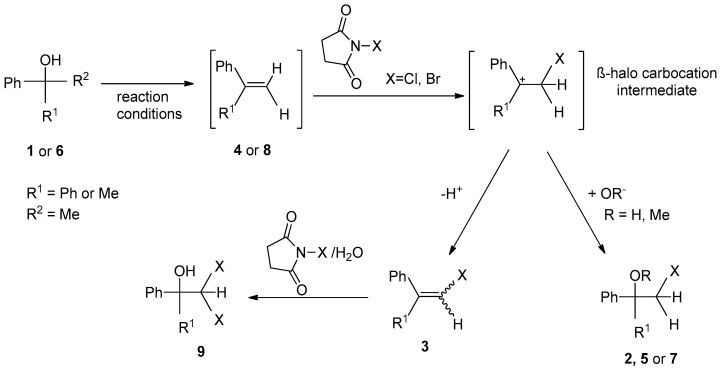
Plausible reaction pathway for direct halogenation of phenyl-alkyl substituted tertiary alcohols to vicinal halohydrins using *N*-halosuccinimides.

On the basis of the presented results we could propose a plausible reaction pathway for the transformation of phenyl-alkyl substituted tertiary alcohols to vicinal halohydrins using *N*-halosuccinimides as the halogenating reagents (Scheme 2). We suppose that the first step of the reaction is the dehydration of the alcohol under the described reaction conditions, thus forming the corresponding alkene; in our case 1,1-diphenylethene (**4**) or 2-phenylpropyl-1-ene (**8**), as observed among the products in the isolated crude reaction mixtures. A certain degree of protic conditions are necessary for this first step, since in aprotic solvents no reaction was observed (Table 1, entries 1–4), while the addition of water to aprotic EtOAc readily resulted in dehydration of the alcohol (Table 1, entries 5 and 6). The second step should be the halogenation of the alkene with NXS reagents, known as the halogene-transfer reagents, to electron-rich unsaturated organic compounds following the electrophilic process which includes the formation of the most stable β-halosubstituted carbocation intermediates. These intermediates could lose a proton and result in the formation of halogen-substituted alkenes, **3** or, in the presence of a sufficient concentration of hydroxy or methoxy nucleophile, it could collapse with them, thus forming vicinal halohydrins, **2** and **7** or halomethoxy analoques **5** respectively. In the cases of performing reactions of 1,1-diphenyl ethanol in water, the formation of vicinal dihalohydrins **9** were also observed in approximately 10% relative yield. The formation of dihalohydins is the consequence of further halohydroxy transformation of the addition-elimination product **3**.

## 3. Materials and Methods

All alcohol substrates were commercially available and were used without further purification. Reactions were carried out in 10 mL round-bottom flasks. All reactions were monitored by thin layer chromatography (TLC) (using silica gel/TLC cards, DC-Alufolien-Kieselgel (Fluka, Sigma-Aldrich, St. Louis, MO, USA). (mobile phase: hexane/ethyl acetate) and visualized by UV lamp (254 nm) and KI (0.1 M) test. Column chromatography (CC) was performed using silica gel Kieselgel 60 (Fluka, Sigma-Aldrich, particle size: 0.063–0.200 mm). Spectroscopic methods: nuclear magnetic resonance Varian INOVA 300 NMR instrument, recorded at National Institute of Chemistry, Ljubljana, Slovenia ^1^H: at 303.0 MHz, ^13^C: at 76.2 MHz using CDCl_3_ as the solvent with SiMe_4_ (TMS) as an internal reference and melting points (open capillary tube methodology; uncorrected, by Buchi 535 equipment at the Jožef Stefan Institute, Ljubljana, Slovenia) were used for identification and structure elucidation.

### General Procedure for Vicinal Halogenation of Tertiary Benzyl Alcohols Using NXS on mmol Scale

A solvent (5 mL) was placed in a 10 mL round bottom-flask equipped with a magnetic stirrer under reflux, NXS (1.1 mmol) was then added and, after being dissolved, the substrate (1 mmol) was added and the solution was stirred for 4–24 h. The progress of the reaction mixture was monitored by TLC, whilst the consumption of NXS was tested by KI (0.1 M). Upon completion of the reaction, the crude reaction mixture was cooled down at room temperature, diluted with EtOAc (15 mL), washed with aqueous Na_2_S_2_O_3_ (6 mL), NaHCO_3_ (6 mL), and water (10 mL), and dried over anhydrous Na_2_SO_4_. The solvent was evaporated under reduced pressure and the crude product obtained was analyzed by ^1^H-NMR. The pure final products were obtained after flash chromatography, column chromatography or preparative thin layer chromatography. The characterization and NMR spectra of the isolated products can be found in the Supplementary Materials.

## 4. Conclusions

The assumption that tertiary benzyl alcohols bearing at least one vicinal hydrogen atom could be directly transformed to the vicinal halohydrins **2a**–**b** or **7a**–**b** using *N*-halosuccinimides was verified on 1,1-diphenylethanol (**1**) and 2-phenyl-2-propanol (**6**) as the basic model compounds. In both cases the efficient and selective one pot synthesis to the corresponding vicinal chlorohydrins **2a** or **7a** and bromohydrins **2b** or **7b** was achieved using NCS or NBS respectively, in aqueous reaction media. However, in the case of the more hydrophobic 1,1-diphenylethanol (**1**), the addition of sodium dodecyl sulphate in the amounts around its critical micelle concentration was found to be essential in order to considerably enhance the yield of the transformation. When acetic acid was used as the reaction media the addition-elimination process took place, resulting in the selective formation of 2-chloro-1-1-diphenylethene (**3a**) or 2-bromo-1-1-diphenylethene (**3b**), while under the same conditions a complex reaction course took place in the case of treatment of 2-phenyl-2-propanol (**6**).

## Figures and Tables

**Table 1:**
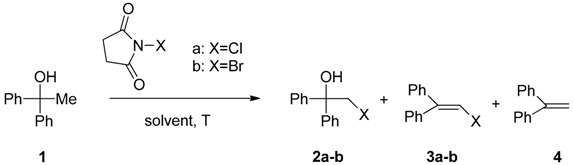
Transformation of 1,1-diphenylethanol **1** with *N*-chlorosuccinimide (NCS) and *N*-bromosuccinimide (NBS) in organic solvents ^a^.

Entry	X	Solvent	Reaction Conditions	Conversion ^b^ (%) of 1	Relative Distribution of Products ^b^ (%)		
					2a–b	3a–b	4
1 ^c^	Cl	Solkane^TM^ 365	reflux, 18 h	/	/	/	/
2 ^c^	Br	Solkane^TM^ 365	reflux, 18 h	/	/	/	/
3	Cl	EtOAc	reflux, 18 h	/	/	/	/
4	Br	EtOAc	reflux, 18 h	32	3	5	24
5 ^d^	Cl	EtOAc/H_2_O	75–80 °C, 18 h	100	/	/	100
6 ^d^	Br	EtOAc/H_2_O	75–80 °C, 18 h	100	/	/	100
7	Cl	2-MeTHF	reflux, 18 h	81	2	1	78
8	Br	2-MeTHF	reflux, 18 h	89	7	/	82
9	Cl	AcOH	reflux, 24 h	100	/	98	2
10	Br	AcOH	reflux, 24 h	100	/	100	/
11	Cl	AcOH	reflux, 4 h	100	/	96	4
12	Br	AcOH	reflux, 4 h	100	/	100	/
13	Cl	AcOH	70–75 °C, 4 h	100	/	100	/
14	Br	AcOH	70–75 °C, 4 h	100	/	60	40
15	Cl	AcOH	40 °C, 4 h	/	/	/	/
16	Br	AcOH	40 °C, 4 h	/	/	/	/

^a^ Reaction conditions: 1,1-diphenylethanol (**1**) (0.5–1 mmol), *N*-halosuccinimide (NXS) (0.55–1.1 mmol), solvent (5 mL); ^b^ determined from ^1^H-NMR spectra of isolated crude reaction mixtures; ^c^ reaction components were not completely soluble. ^d^ EtOAc (4 mL)/H_2_O (1 mL).

**Table 2:**
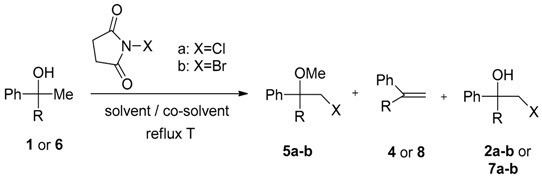
Transformation of 1,1-diphenylethanol (**1**) or 2-phenyl-2-propanol (**6**) with NCS and NBS in methanol, acetic acid, aqueous media and their mixtures ^a^.

Entry	R	X	Solvent/Co-Solvent	Conversion ^b^ (%) of 1	Relative Distribution ^b^ of Products (%)		
					5a–b	4 or 8	2a–b or 7a–b
1	Ph	Cl	MeOH (5 mL)	100	73	23	/ ^c^
2	Ph	Br	MeOH (5 mL)	100	40	60	/
3	Ph	Cl	MeOH (5 mL)/H_2_O (20 mmol)	98	60	30	5 ^c^
4	Ph	Br	MeOH (5 mL)/H_2_O (20 mmol)	100	13	84	3
5	Ph	Cl	MeOH (2.5 mL)/H_2_O (2.5 mL)	100	43	25	29
6	Ph	Br	MeOH (2.5 mL)/H_2_O (2.5 mL)	100	9	74	7 ^c^
7	Me	Cl	AcOH (2.5 mL)/H_2_O (2.5 mL)	100	/	/	93 ^d^
8	Me	Br	AcOH (2.5 mL)/H_2_O (2.5 mL)	100	/	/	95 ^e^
9	Ph	Cl	H_2_O (5 mL)	14	/	/	14
10	Ph	Br	H_2_O (5 mL)	72	/	/	54 ^f^
11	Me	Cl	H_2_O (5 mL)	100	/	/	100
12	Me	Br	H_2_O (5 mL)	96	/	44	52

^a^ Reaction conditions: alcohol **1** or **6** (1 mmol), NXS (1.1 mmol), solvent, under reflux, 4–22 h; ^b^ determined from ^1^H-NMR spectra of isolated crude reaction mixtures; ^c^ trace amounts of other products (3%–10%); ^d^ isolated pure product; ^e^ isolated crude product; ^f^ 18% of 2,2-dibromo-1,1-diphenylethanol was also formed.

**Table 3:**
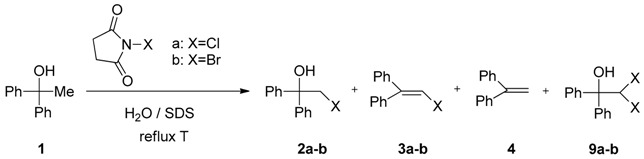
The course of the reaction of 1,1-diphenylethanol (**1**) with *N*-halosuccinimide (NXS) in aqueous media in the presence of sodium dodecyl sulphate (SDS) amphiphile ^a^.

Entry	X	H_2_O/SDS	Conversion ^b^ (%) of 1	Relative Distribution of Products ^b^ (%)			
				2a–b	3a–b	4	9a–b
1	Cl	SDS (c = 0.0008 M)	82	47	11	24	/
2	Br	SDS (c = 0.0008 M)	95	53	12	24	6
3	Cl	SDS (c = 0.001 M)	95	47	10	32	6
4	Br	SDS (c = 0.001 M)	96	49	10	27	10
5	Cl	SDS (c = 0.003 M)	100	72	8	20	/
6	Br	SDS (c = 0.003 M)	100	56	5	28	12
7	Cl	SDS (c = 0.008 M)	100	86	7	7	/
8	Br	SDS (c = 0.008 M)	100	67	5	17	11
9	Cl	SDS (c = 0.05 M)	100	89	4	7	/
10	Br	SDS (c = 0.05 M)	100	70	/	23	7

^a^ Reaction conditions: 1,1-diphenylethanol (**1**) (1 mmol), NXS (1.1 mmol), solvent (5 mL), under reflux, 7 h; ^b^ determined from ^1^H-NMR spectra of isolated crude reaction mixtures.

## Supplementary Materials

The following are available online at www.mdpi.com/1420-3049/21/10/1325/s1, S1. General information, S2. General procedure for vicinal halogenation of tertiary benzyl alcohols, S3. Characterization data of isolated final products, S4. ^1^H-NMR and ^13^C-NMR spectra of isolated final products, S5. References.
