# Supplementary material for: Transformation of Tertiary Benzyl Alcohols into the Vicinal Halo-Substituted Derivatives Using N-Halosuccinimides
Source: Molecules. 2016 Oct 2;21(10):1325. doi: 10.3390/molecules21101325 (PMC6273677; doi:10.3390/molecules21101325)
Supplement: Supplementary file 1 [file molecules-21-01325-s001.pdf]

Supplementary Materials: Transformation of Tertiary Benzyl Alcohols into the Vicinal Halo-Substituted Derivatives Using *N*-Halosuccinimides

Njomza Ajvazi and Stojan Stavber

S1. General Information S2
S2. General Procedure for Vicinal halogenation of tertiary benzyl alcohols S2
S3. Characterization data of isolated final products S2
S4. ^{1}H-NMR and ^{13}C-NMR spectra of isolated final products S4
References S11

S1. General Information

All alcohol substrates were commercially available and were used without further purification. Reactions were carried out in 10 mL round-bottom flasks. All reactions were monitored by thin layer chromatography (TLC) (using silica gel/TLC cards,DC-Alufolien-Kieselgel (Fluka, Sigma-Aldrich, St. Louis, MO, USA), (mobile phase: hexane/ethyl acetate) and visualized by UV lamp (254 nm) and KI (0.1 M) test. Column chromatography (CC) was performed using silica gel Kieselgel 60 (Fluka, Sigma-Aldrich) (particle size: 0.063–0.200 mm). Spectroscopic methods: nuclear magnetic resonance (Varian INOVA 300 NMR instrument, recorded at National Institute of Chemistry, Ljubljana, Slovenia, ^{1}H: at 303.0 MHz, ^{13}C: at 76.2 MHz) using $CDCl_3$ as the solvent with $SiMe_4$ (TMS) as an internal reference and melting points (open capillary tube methodology; uncorrected, by Buchi 535 equipment at the Jožef Stefan Institute, Ljubljana, Slovenia) were used for identification and structure elucidation.

S2. General Procedure for Vicinal Halo-Substituted Derivatives sing NXS on mmol Scale

A solvent (5 mL) was placed in a 10 mL round bottom-flask equipped with a magnetic stirrer under reflux, NXS (1.1 mmol) was then added and, after being dissolved, the substrate (1 mmol) was added and the solution was stirred for 4–24 h. The progress of the reaction mixture was monitored by TLC, whilst the consumption of NXS was tested by KI (0.1 M). Upon completion of the reaction, the crude reaction mixture was cooled down at room temperature, diluted with EtOAc (15 mL), washed with aqueous $Na_2S_2O_3$ (6 mL), $NaHCO_3$ (6 mL), and water (10 mL), and dried over anhydrous Na_2SO_4. The solvent was evaporated under reduced pressure and the crude product obtained was analyzed by ^{1}H-NMR. The pure final products were obtained after flash chromatography, column chromatography or preparative thin layer chromatography.

S3. Characterization Data of Isolated Final Products

Table S1. 2-Chloro-1,1-diphenylethanol (**2a**) [1].

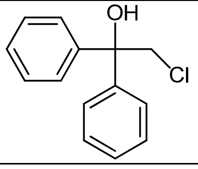	**$C_{14}H_{13}ClO$ (Mr = 232.71)**
Reaction conditions	1 mmol 1,1-diphenylethanol **1** (198.1 mg), 1.1 mmol NCS (146.8 mg), 0.008 M SDS (11.5 mg), H_2O (5 mL), 95–100 °C, 7 h stirring 1000 rpm
Purification	CC (SiO_2, EtOAc/hexane 9:1)
Yield	186 mg (80%), colourless oil
^{1}H-NMR (300 MHz, $CDCl_3$)	δ = 7.45–7.25 (m, 10H), 4.18 (s, 2H), 3.16 (s, 1H)
^{13}C-NMR (76 MHz, $CDCl_3$)	δ = 143.4, 128.5, 127.8, 126.5, 78.0, 53.3

Table S2. 2-Bromo-1,1-diphenylethanol (**2b**) [2].

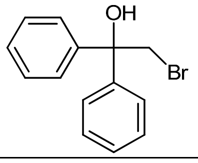	**$C_{14}H_{13}BrO$ (Mr = 277.16)**
Reaction conditions	1 mmol 1,1-diphenylethanol **1** (198.1 mg), 1.1 mmol NBS (195.6 mg), 0.05M SDS (72 mg), H_2O (5 mL), 95–100 °C, 7 h stirring 1000 rpm
Purification	TLC-preparative (EtOAc/hexane 9:1)
Yield	164 mg (59%), yellow oil
^{1}H-NMR (300 MHz, $CDCl_3$)	δ = 7.54–7.20 (m, 10H), 4.11 (s, 2H), 3.10 (s, 1H)
^{13}C-NMR (76 MHz, $CDCl_3$)	δ = 143.4, 128.5, 127.8, 126.4, 77.3, 44.1

Table S3. (2-Chloroethene-1,1-diyl)dibenzene (**3a**) [3].

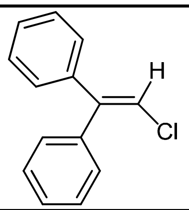	**$C_{14}H_{11}Cl$ (Mr = 214.69)**
Reaction conditions	1 mmol 1,1-diphenylethanol **1** (198.1 mg), 1.1 mmol NCS (146.8 mg), CH_3COOH (5 mL), 115–117 °C, 4 h stirring 400 rpm
Purification	Crystalization (hexane)
Yield	173 mg (80%), white crystals
Mp.	39–40 °C, (lit.[3] 39–40 °C)
^{1}H-NMR (300 MHz, $CDCl_3$)	δ = 7.41–7.18 (m, 10H), 6.58 (s, 1H)
^{13}C-NMR (76 MHz, $CDCl_3$)	δ = 144.0, 140.3, 137.7, 130.0, 128.6, 128.3, 128.2, 128.1, 127.9, 116.0

Table S4. (2-Bromoethene-1,1-diyl)dibenzene (**3b**) [4].

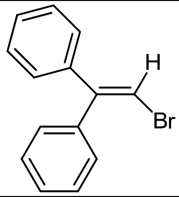	**$C_{14}H_{11}Br$ (Mr = 259.14)**
Reaction conditions	1 mmol 1,1-diphenylethanol **1** (198.1 mg), 1.1 mmol NBS (195.6 mg), CH_3COOH (5 mL), 115–117 °C, 4 h stirring 400 rpm
Purification	FCH (SiO_2, EtOAc); white crystals
Yield	250 mg (97%), white crystals
Mp.	47–49 °C, (Ref. [4] 48–50 °C)
^{1}H-NMR (300 MHz, $CDCl_3$)	δ = 7.42–7.18 (m, 10H), 6.77 (s, 1H)
^{13}C-NMR (76 MHz, $CDCl_3$)	δ = 147.0, 140.9, 139.2, 129.8, 128.6, 128.4, 128.3, 128.1, 127.8, 105.3

Table S5. (2-chloro-1-methoxyethane-1,1-diyl)dibenzene (**5a**) [5].

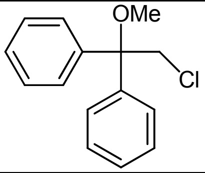	**$C_{15}H_{15}ClO$ (Mr = 246.73)**
Reaction conditions	1 mmol 1,1-diphenylethanol **1** (198.1 mg), 1.1 mmol NCS (146.8 mg), CH_3OH (5 mL), 60–70 °C, 22 h stirring 400 rpm
Purification	CC (SiO_2, EtOAc/hexane 9:1)
Yield	160 mg (65%), white solid
Mp.	46–48 °C, (Ref. [5] 47–49 °C)
^{1}H-NMR (300 MHz, $CDCl_3$)	δ = 7.38–7.25 (m, 10H), 4.34 (s, 2H), 3.18 (s, 3H)
^{13}C-NMR (76 MHz, $CDCl_3$)	δ = 142.6, 128.2, 127.6, 127.3, 82.0, 50.9, 48.8

Table S6. (2-bromo-1-methoxyethane-1,1-diyl)dibenzene (**5b**) [6].

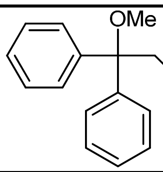	**$C_{15}H_{15}BrO$ (Mr = 291.18)**
Reaction conditions	1 mmol 1,1-diphenylethanol **1** (198.1 mg), 1.1 mmol NBS (195.6 mg), CH_3OH (5 mL), 60–70 °C, 22 h stirring 400
Purification	CC (SiO_2, EtOAc/hexane 9:1)
Yield	102 mg (35%), colourless oil
^{1}H-NMR (300 MHz, $CDCl_3$)	δ = 7.38–7.22 (m, 10H), 4.23 (s, 2H), 3.16 (s, 3H) rpm
^{13}C-NMR (76 MHz, $CDCl_3$)	δ = 142.8, 128.2, 127.5, 127.2, 81.3, 50.7, 38.8

Table S7. 1-chloro-2-phenylpropan-2-ol (**7a**) [1].

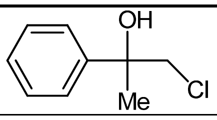	**$C_9H_{11}ClO$ (Mr = 170.64)**
Reaction conditions	1 mmol 2-phenylpropan-2-ol **6** (136.1 mg), 1.1 mmol NCS (146.8 mg), CH_3COOH (5 mL), 115–117 °C, 4 h stirring 400 rpm
Purification	FCH (SiO_2, EtOAc)
Yield	159 mg (93%), colourless oil
^{1}H-NMR (300 MHz, $CDCl_3$)	δ = 7.50–7.25 (m, 5H), 3.86–3.73 (m, 2H), 2.60 (s, 1H), 1.64 (s, 3H)
^{13}C-NMR (76 MHz, $CDCl_3$)	δ = 144.3, 128.5, 127.6, 125.1, 73.9, 55.5, 27.4

S4. ^{1}H-NMR and ^{13}C-NMR Spectra of Isolated Final Products

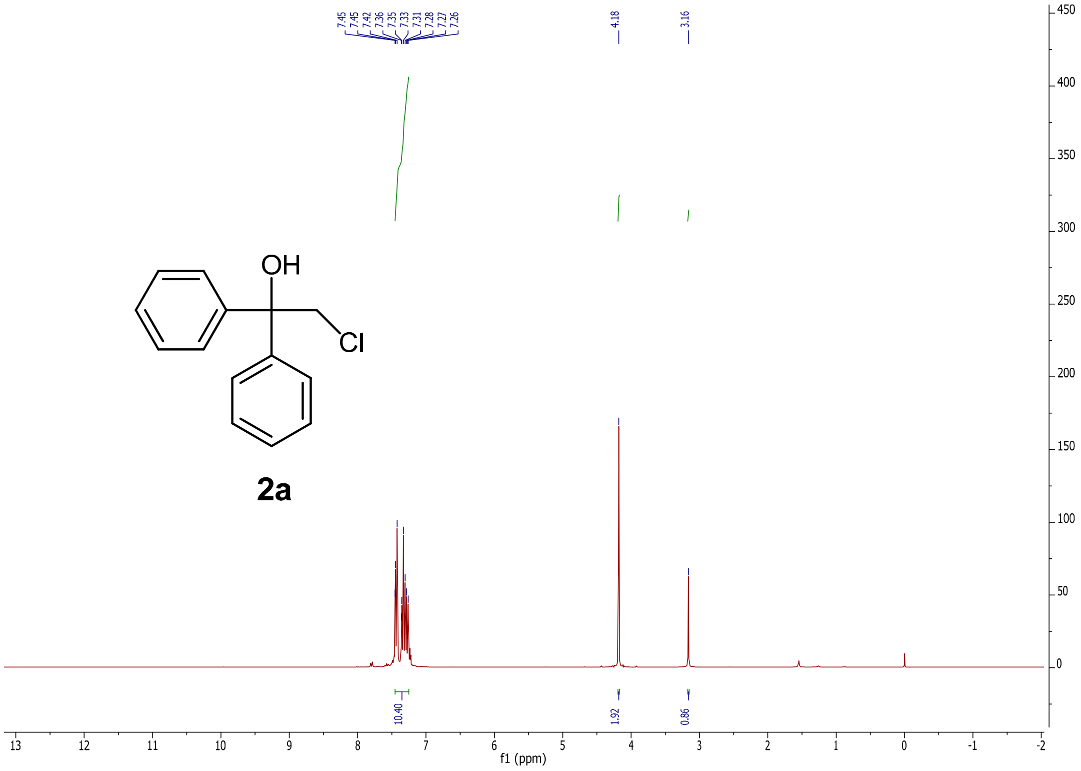

Figure S1. ^{1}H-NMR spectrum of compound **2a**.

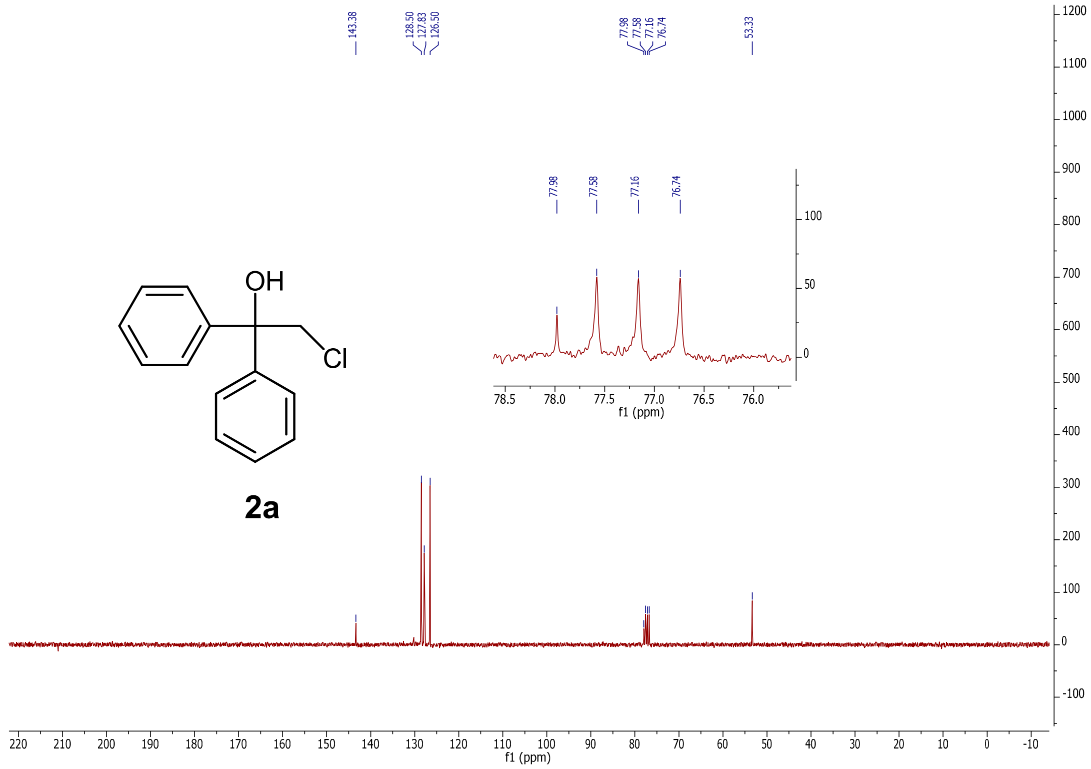

Figure S2. ^{13}C-NMR spectrum of compound **2a**.

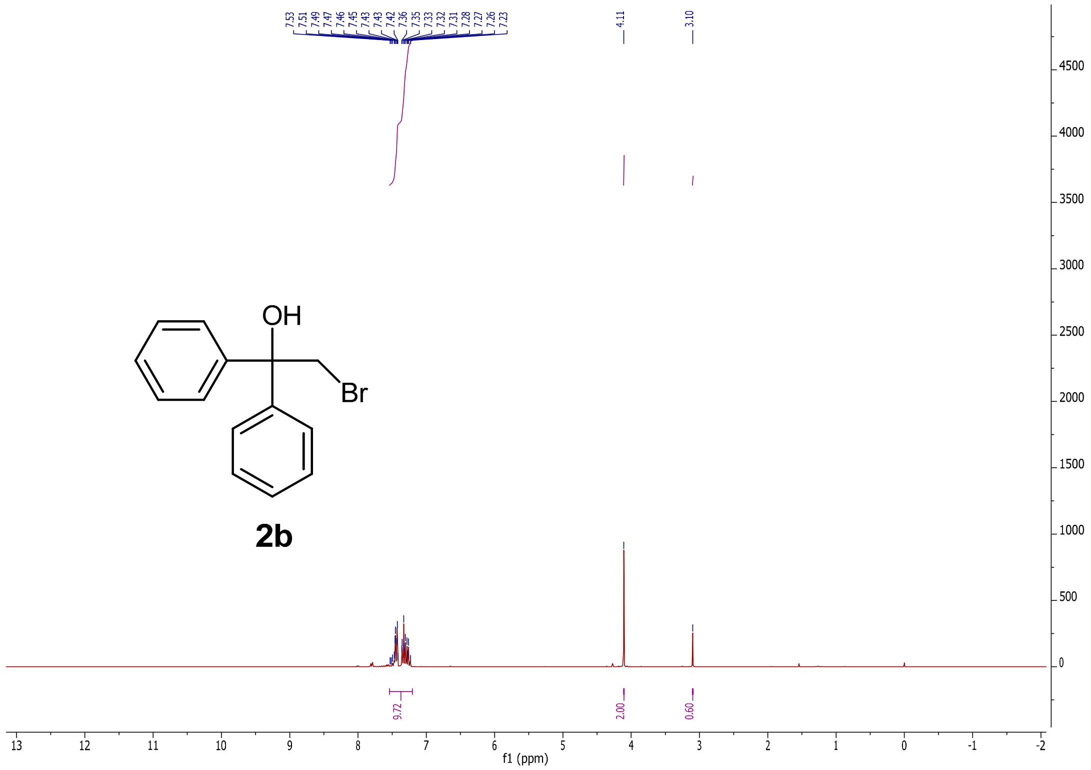

Figure S3. ^{1}H-NMR spectrum of compound **2b**.

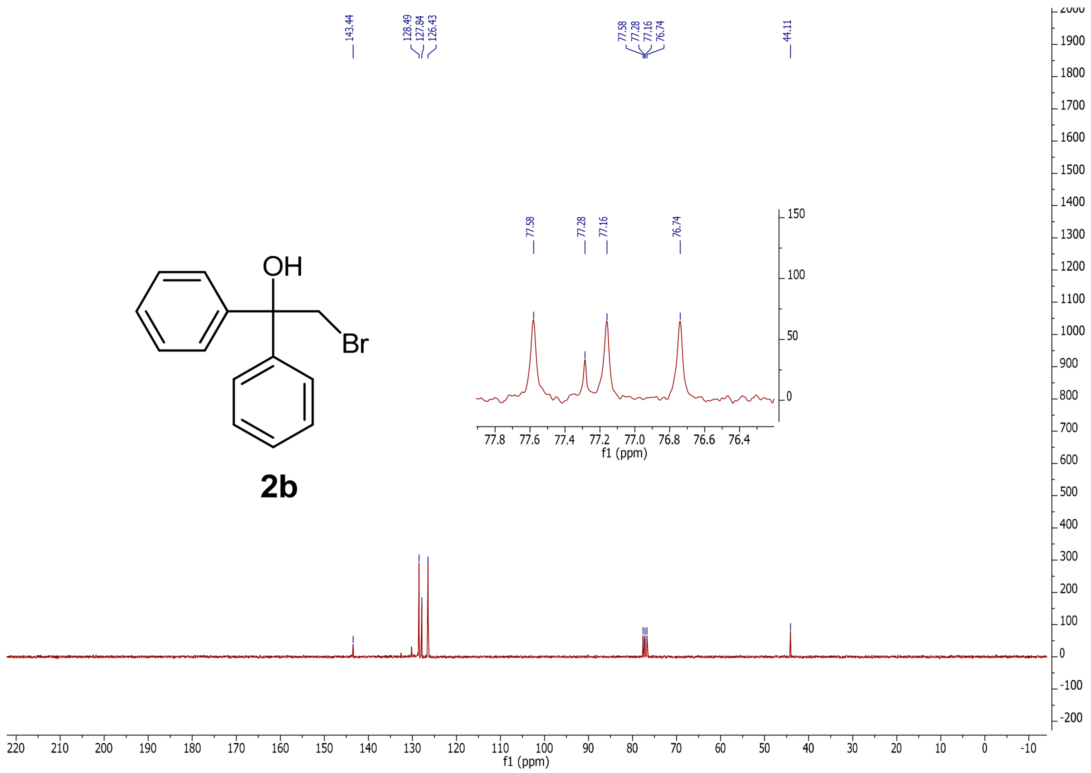

Figure S4. ^{13}C-NMR spectrum of compound **2b**.

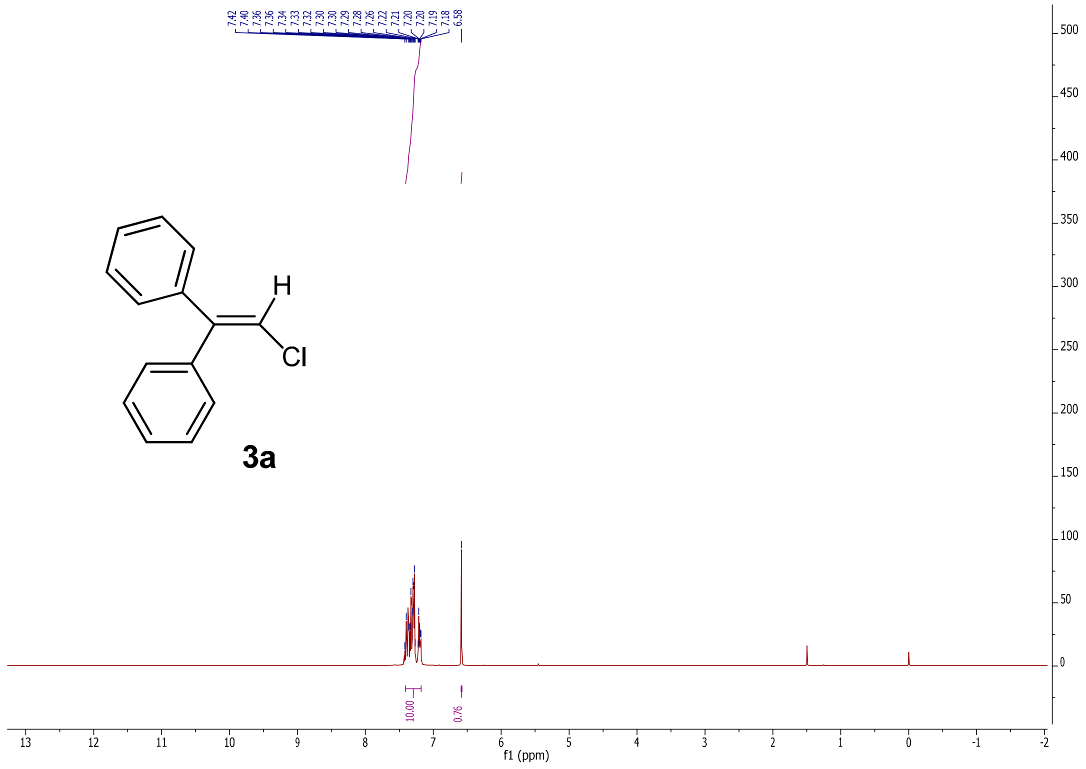

Figure S5. ^{1}H-NMR spectrum of compound **3a**.

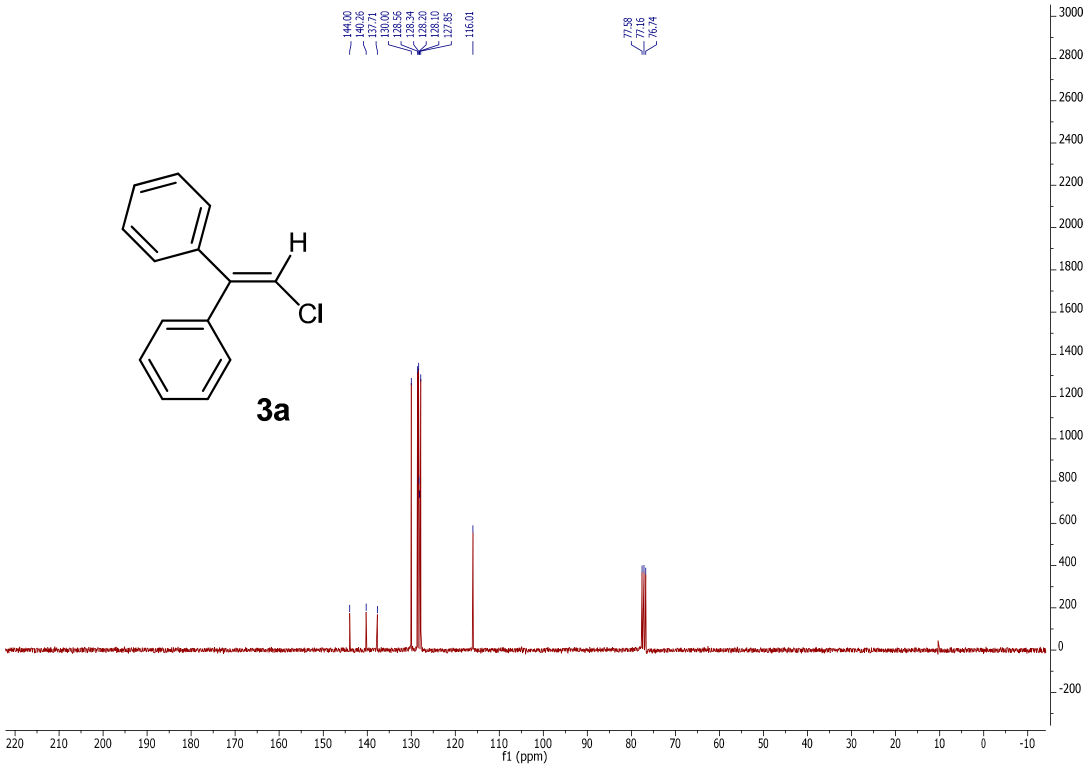

Figure S6. ^{13}C-NMR spectrum of compound **3a**.

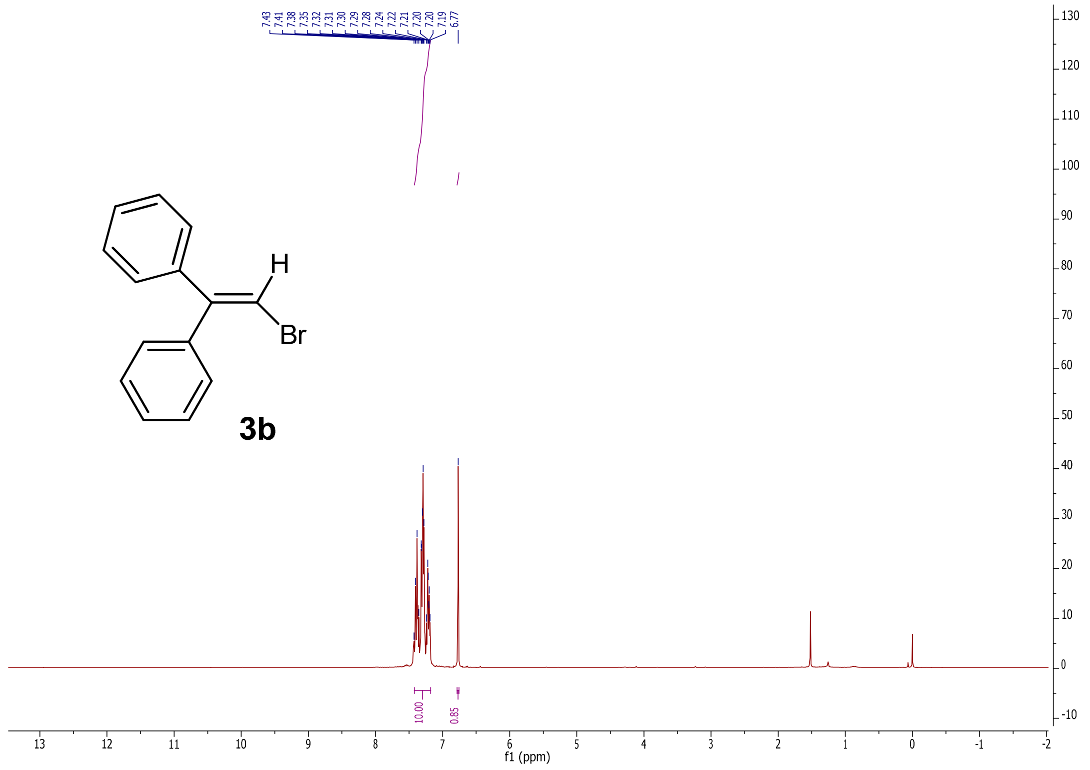

Figure S7. ^{1}H-NMR spectrum of compound **3b**.

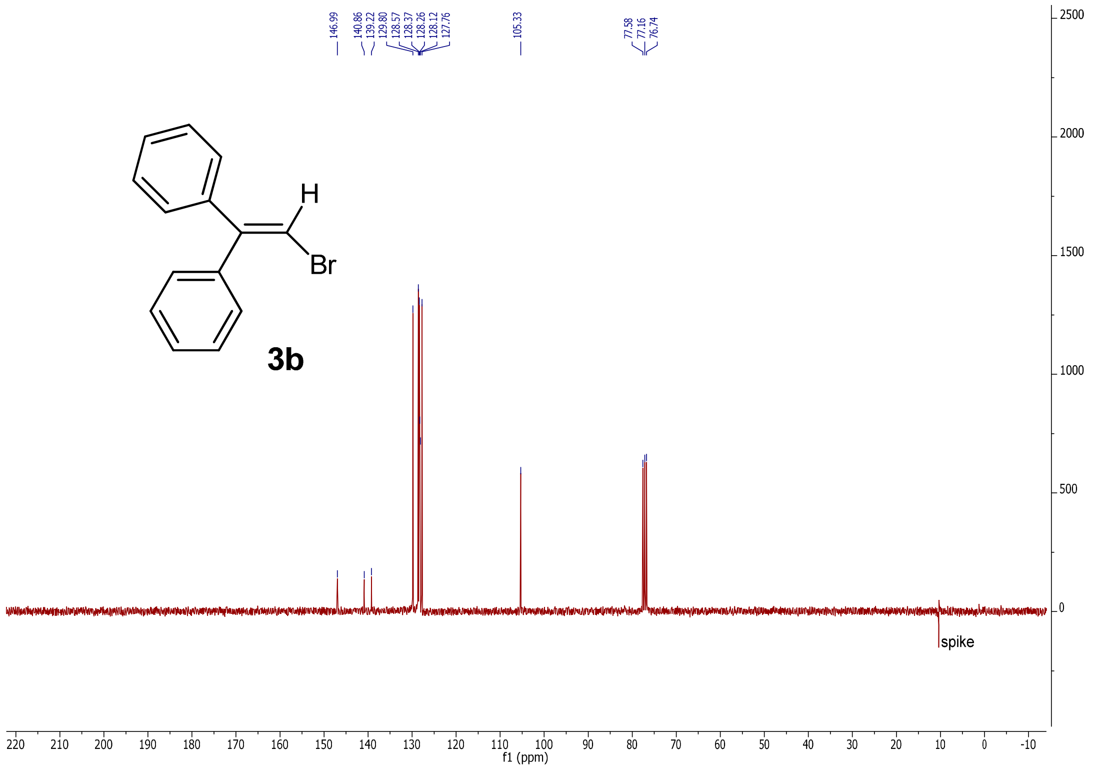

Figure S8. ^{13}C-NMR spectrum of compound **3b**.

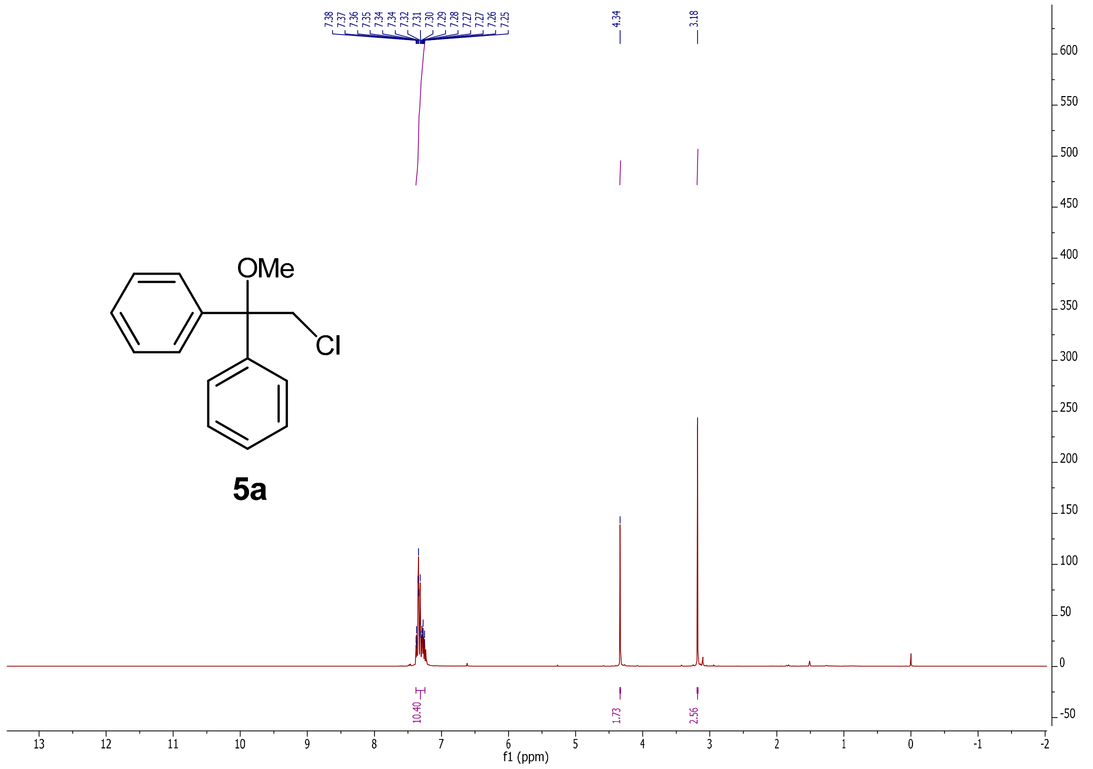

Figure S9. ^{1}H-NMR spectrum of compound **5a**.

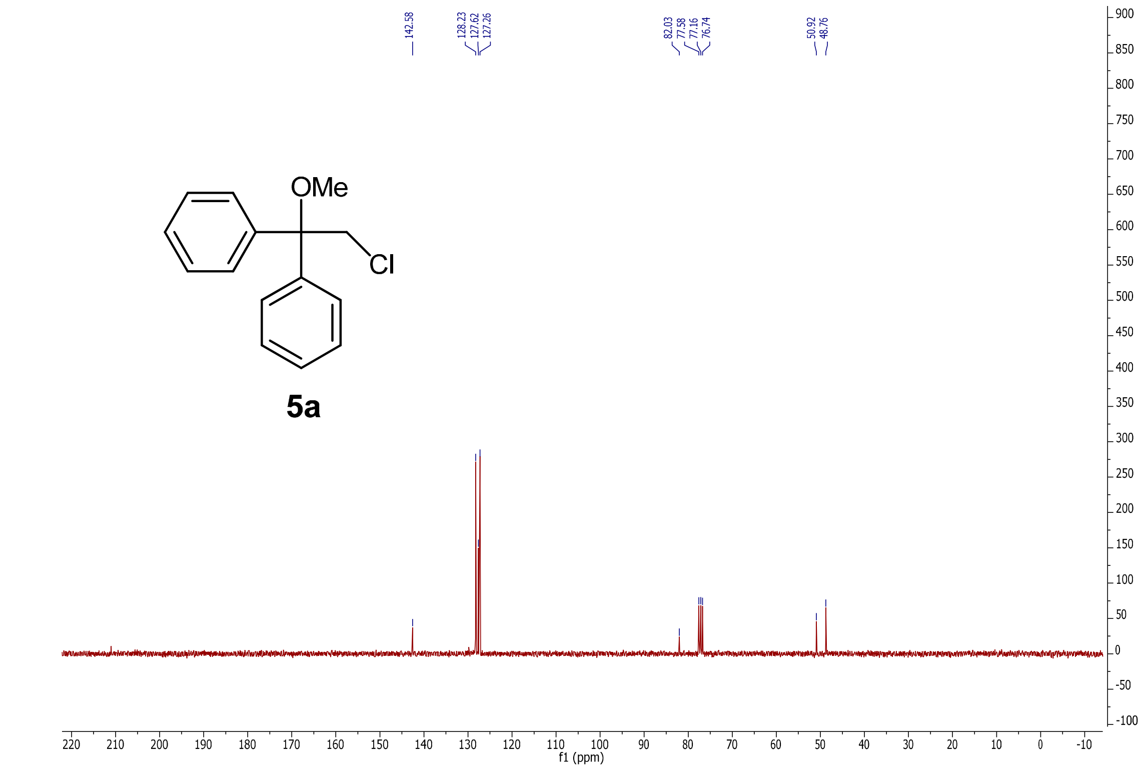

Figure S10. ^{13}C-NMR spectrum of compound **5a**.

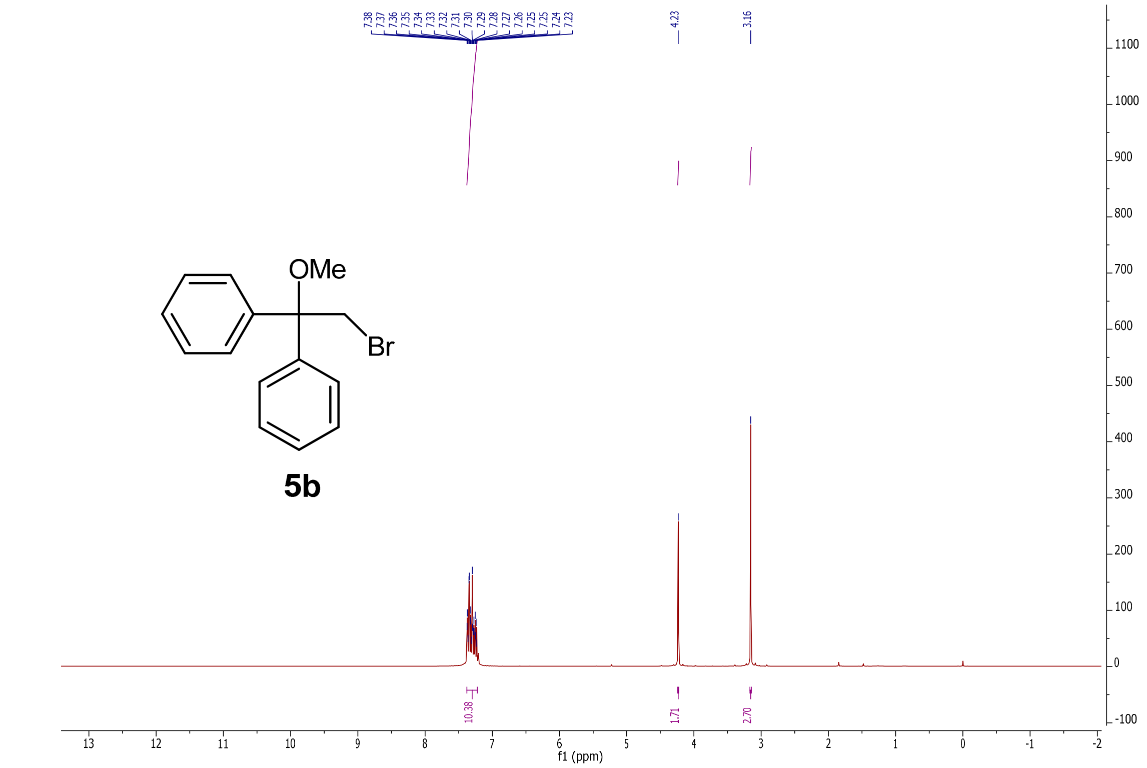

Figure S11. ^{1}H-NMR spectrum of compound **5b**.

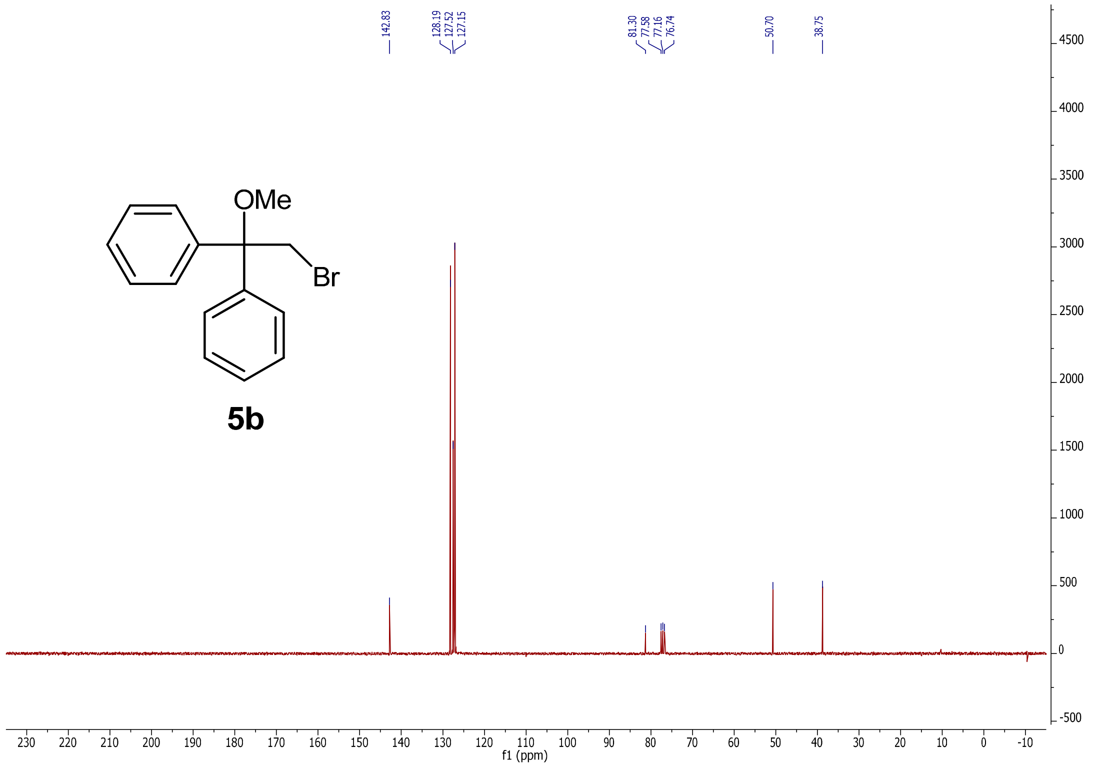

Figure S12. ^{13}C-NMR spectrum of compound **5b**.

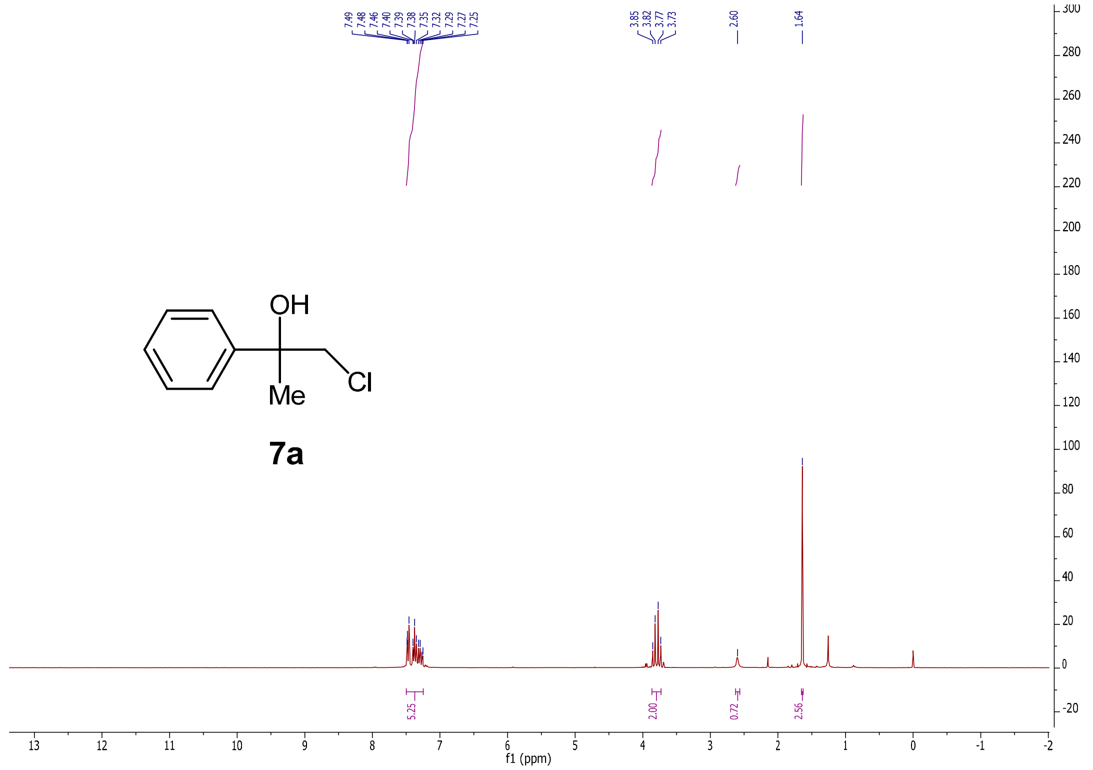

Figure S13. ^{1}H-NMR spectrum of compound **7a**.

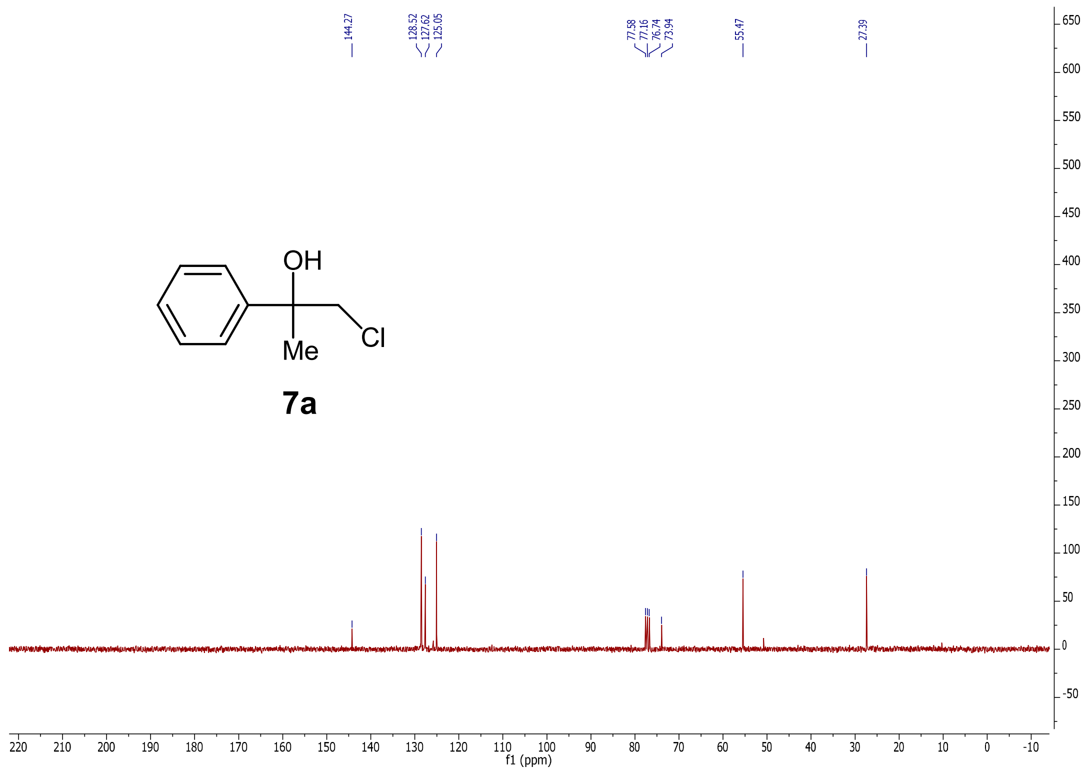

Figure S14. ^{13}C-NMR spectrum of compound **7a**.

References

1. Degennaro, L.; Fanelli, F.; Giovine, A.; Luisi, R. External Trapping of Halomethyllithium Enabled by Flow Microreactors. *Adv. Synth. Catal.* **2015**, *357*, 21–27.
2. Yu, L.; Ren, L.; Yi, R.; Wu, Y.; Chen, T.; Guo, R. Palladium-Catalyzed Reaction of Olefins with $PhI(OAc)_2$-TBAB System: An Efficient and Highly Selective Bisfunctionalization Strategy. *Synlett* **2011**, *2011*, 579–581.
3. Takeda, T.; Endo, Y.; Reddy, A.C.S.; Sasaki, R.; Fujiwara, T. Transformation of ketones into 1-chloro and 1,1-dichloro-1-alkenes by means of a polychloromethane-titanocene(II) system. *Tetrahedron* **1999**, *55*, 2475–2486.
4. Podgoršek, A.; Eissen, M.; Fleckenstein, J.; Stavber, S.; Zupan, M.; Iskra, J. Selective aerobic oxidative dibromination of alkenes with aqueous HBr and sodium nitrite as a catalyst. *Green Chem.* **2009**, *11*, 120–126.
5. Yusubov, M.S.; Yusubova, R.Y.; Filimonov, V.D.; Ki-Whan, C. Iodosobenzene Dichloride, a Convenient Reagent for Chloro- and Iodomethoxylation of Double and Triple Bonds. *Russ. J. Org. Chem.* **2002**, *38*, 902–904.
6. Ziegler, K.; Richter, K.; Schnell, B., Über echte Derivate des Diphenyl-styryl-methans. 6. Mitteilung über mehrfach aryl-substituierte Vinylcarbinole und ihre Derivate. *Liebigs Ann.* **1925**, *443*, 161–180.
